# Unusual manifestation of genital cutaneous leishmaniasis in an immunocompetent patient from São Paulo, Brazil: A case report

**DOI:** 10.1590/0037-8682-0514-2020

**Published:** 2021-03-22

**Authors:** Luiza Campos Reis, José Angelo Lauletta Lindoso, Beatriz Julieta Celeste, Lucia Maria Almeida Braz, Eduardo Milton Ramos-Sanchez, Edite Hatsumi Yamashiro-Kanashiro, Hiro Goto, Luiza Keiko Matsuka Oyafuso

**Affiliations:** 1 Universidade de São Paulo, Faculdade de Medicina, Instituto de Medicina Tropical de São Paulo, São Paulo, SP, Brasil.; 2 Secretaria de Saúde do Estado de São Paulo, Instituto de Infectologia Emílio Ribas, São Paulo, SP, Brasil.; 3 Universidade de São Paulo, Faculdade de Medicina, Departamento de Moléstias Infecciosas e Parasitárias, São Paulo, SP, Brasil.; 4 Universidad Nacional Toribio Rodriguez de Mendoza de Amazonas, Facultad de Ciencias de La Salud, Departamento de Salud Publica, Chachapoyas, Peru.; 5 Universidade de São Paulo, Faculdade de Medicina, Departamento de Medicina Preventiva, São Paulo, SP, Brasil.

**Keywords:** Cutaneous leishmaniasis, Diagnosis, Genital lesion

## Abstract

A 31-year-old male patient developed an ulcer on the glans penis that evolved for three months without healing. We diagnosed it as leishmaniasis using polymerase chain reaction. No immunosuppression or associated diseases were observed. The patient was treated with meglumine antimoniate that cured the lesion in a month post-treatment. Here, we report this case of cutaneous leishmaniasis lesion at the unusual location of glans penis in an immunocompetent individual. The lesion likely developed due to the bite of a vector, highlighting the need for considering cutaneous leishmaniasis among differential diagnosis of sexually transmitted diseases in areas endemic for leishmaniasis.

## INTRODUCTION

Cutaneous leishmaniasis (CL) is a vector-transmitted protozoan disease caused by different species of *Leishmania*. In Brazil, CL is mainly caused by the species *Leishmania (Viannia) braziliensis*, *L. (V.) guyanensis*, *L. (Leishmania) amazonensis* ^1^, and approximately 238,000 cases of CL were reported between 2007 and 2018. In the state of São Paulo during the same period, 4,159 cases were reported, of which 55% were autochthonous cases^2^. 

Its clinical manifestations vary from localized, disseminated, and diffuse cutaneous forms to severely mutilating mucocutaneous lesions^1^. The initial lesion is at the site of the vector bite in the form of a macule; after two weeks to three months, it may present as a small, pruritic, erythematous papule, and/or nodule and may involve draining lymph nodes. The lesion may resolve spontaneously or develop into a characteristic ulcer; some lesions may develop into other chronic forms. Mucocutaneous leishmaniasis is the most aggressive and mutilating form characterized by infiltration, ulceration, and destruction of mucous membranes and cartilage, and occurs years after the onset of CL. The lesions of CL are localized mainly in exposed body parts, such as the arm, leg, and face. Nasal and oral mucosae are the most affected in the mucocutaneous form. Its clinical manifestations depend on the species of the parasite, the vector, and the genetic and immunological constitution of the host^1^ ^,^ ^3^. The genital region is rarely a primary site. In immunosuppressed patients, however, studies have described genital involvement as a consequence of the spread of the parasite from the initial lesion^4^ ^,^ ^5^. 

The etiological diagnosis of CL is sometimes difficult, and therefore, epidemiological, clinical, and histopathological findings should be considered. Concerning clinical manifestations, the lesions present suggestive characteristics but may vary considerably. They may resemble ulcerated or non-ulcerated lesions from other diseases, such as Virchow’s hanseniasis, paracoccidioidomycosis, tropical ulcers, syphilis, and cutaneous tuberculosis, thus requiring its differential diagnosis. Moreover, conventional diagnostic methods are limited in their efficiency, necessitating a combination of different approaches to diagnose leishmaniasis^6^. 

Here, we report a case of CL with a lesion in the genital area in a patient from São Paulo, who did not exhibit any immunosuppression or associated disease, thus posing a challenge in diagnosis.

## CASE REPORT

**FIGURE 1:**
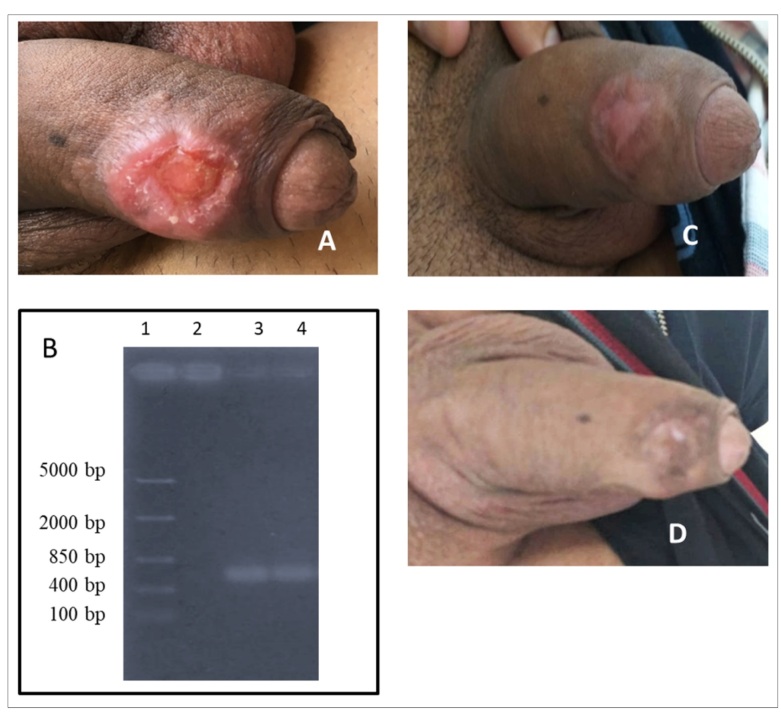
**(A)** Ulcer of glans penis; **(B)** Polymerase chain reaction (PCR) amplification of the kDNA (750 bp) of *Leishmania* subgenus *Viannia* in biopsy sample obtained from patient with cutaneous leishmaniasis (1: DNA ladder (100-5000 bp); 2: *L. amazonensis;* 3: *L. braziliensis*; 4: sample from patient); **(C)** Lesion healed one-month post-treatment. **(D)** Six months post-treatment.

A 31-year-old male patient from Embu das Artes located in the municipality of the Metropolitan Region of São Paulo, in the state of São Paulo in Brazil sought an infectious disease consultation at the Instituto de Infectologia Emílio Ribas. He presented with an ulcer on the glans penis that evolved without healing for three months. The patient reported that he did not travel to other regions recently or perform outdoor activities and that he worked as a salesman in the city but not in rural areas. He had initially noticed a small lesion with raised border without pain that progressively increased in size over few weeks. Clinical examination revealed an ulcer with elevated borders and granulomatous contents (Figure 1A). The lesion measured 3.0 × 2.5 cm in dermatological examination. It was not associated with systemic symptoms, such as fever, weight loss, hepatosplenomegaly, or lymphadenopathy. All hematological and biochemical parameters were within the normal range. Initially, the diagnosis was directed to sexually transmitted diseases, but tests for human immunodeficiency virus (HIV) and syphilis were nonreactive. Thus, we tested for CL based on the lesion characteristics and epidemiological data. Table 1 summarizes the tests performed for the diagnosis of CL-histopathology of the lesion biopsy, direct smear and culture in McNeal, Novy, Nicolle medium of the scraped lesion sample, polymerase chain reaction (PCR) of lesion-derived DNA, and serological assays (anti-*Leishmania* enzyme-linked immunosorbent assay [ELISA] and indirect immunofluorescence [IIF]). 

**TABLE 1:** Laboratory test results.

Diagnostic techniques	Results
Direct smear	−
Parasite isolation	−
ELISA (IgG)	+ (1/160)
IIF (IgG)	−
PCR (kDNA)	+

**ELISA:** enzyme-linked immunosorbent assay**; IIF:** indirect immunofluorescence; **PCR:** polymerase chain reaction. **+:** positive; **−:** negative.

Histopathological examination revealed granulomatous dermatosis with diffuse pattern, vasculopathy, and evident plasmacytosis but *Leishmania* amastigotes. It was negative for acid-alcohol-resistant bacilli (BAAR) and fungi. We diagnosed and confirmed leishmaniasis based on the results of PCR for the 750 bp kinetoplast DNA (kDNA) of the *Leishmania* subgenus *(Viannia)* (using the primers B1. 5’−GGGGTTGGTGTAATATAGTGG−3’ and B2. 5’−CTAATTGTGCACGGGGAGG−3’) (Figure 1B)^7^, and the positive anti-*Leishmania* ELISA. The patient was treated with intravenous meglumine antimoniate (20 mg/kg/day) for 20 days. One-month post-treatment, the lesion completely healed (Figure 1C ). The patient was followed up for six months with no signs of relapse (Figure 1D) or any other lesion in other parts of the body and was considered cured. This study was approved by the Conselho Nacional de Ética em Pesquisa (National Council of Ethics on Research - CAAE: 71602417.0.0000.0065).

## DISCUSSION

In CL, the usual clinical presentation is a single cutaneous ulcerative lesion, usually localized in exposed areas of the body accessible to the sandfly^8^. Lesions of CL in the covered areas of the body is unusual; genital lesions are even rarer with only a few reported cases^4^ ^,^ ^5^ ^,^ ^8^ ^-^ ^11^. Studies suggest that genital lesions may result from possible hematogenic dissemination in patients with multiple cutaneous lesions or in immunosuppressed patients^4^ ^,^ ^5^ ^,^ ^8^. Our patient was immunocompetent and had just a single lesion with no history of CL. 

Initially, the diagnosis was focused on sexually transmitted diseases; however, the results were negative for syphilis and HIV. The patient resides in a region with reported cases of leishmaniasis in the state of São Paulo, with the presence of the vector *Lutzomyia intermedia*, the main species related to the transmission of *L. (V.) braziliensis* ^12^ in São Paulo. Based on this epidemiological analysis, the differential diagnosis for CL was considered. The diagnosis was confirmed using PCR for *Leishmania* kDNA and anti-*Leishmania* ELISA. Other parasitological tests (direct microscopic examination of sample aspirates and cultured material) for CL have low sensitivity, as parasites are few or absent in chronic lesions with long evolution periods^1^ ^,^ ^6^. Our results reinforce that CL should be diagnosed using a combination of clinical, epidemiological, and laboratory approaches, with more than one test performed. With accurate tracing of the patient’s whereabouts, we concluded that the lesion on penis was not a secondary lesion but likely developed at the site of the vector bite when the patient reportedly slept naked due to hot weather; notably, this region is surrounded by the remnants of Atlantic forest. 

The present case illustrates that in an area endemic for leishmaniasis, cases with atypical lesions should be investigated for leishmaniasis. 
